# Alamandine alleviated heart failure and fibrosis in myocardial infarction mice

**DOI:** 10.1186/s13062-022-00338-6

**Published:** 2022-09-27

**Authors:** Kun Zhao, Tianhua Xu, Yukang Mao, Xiaoguang Wu, Dongxu Hua, Yanhui Sheng, Peng Li

**Affiliations:** 1 Department of Cardiology, The First Affiliated Hospital of Nanjing Medical University, 300 Guangzhou Road, Nanjing, 210029 Jiangsu China; 2 Department of Cardiology, The Affiliated Suzhou Hospital of Nanjing Medical University, Suzhou Municipal Hospital, Gusu School, Nanjing Medical University, Suzhou, Jiangsu China; 3 Department of Cardiology, Jiangsu Province Hospital, Nanjing, Jiangsu China

**Keywords:** Alamandine, Heart failure, Cardiac fibrosis, Apoptosis, Oxidative stress

## Abstract

Alamandine (Ala) is the newest identified peptide of the renin-angiotensin system and has protective effect on myocyte hypertrophy. However, it is still unclear whether Ala can alleviate heart failure (HF). The aim of this study was to explore the effects of Ala on HF and the related cardiac fibrosis, and to probe the mechanism. HF model was induced by myocardial infarction (MI) in mice. Four weeks after MI, Ala was administrated by intraperitoneal injection for two weeks. Ala injection significantly improved cardiac dysfunction of MI mice in vivo. The cardiac fibrosis and the related biomarkers were attenuated after Ala administration in HF mice in vivo. The increases of collagen I, alpha-smooth muscle actin and transforming growth factor-beta induced by oxygen–glucose deprivation (OGD) in neonatal rat cardiac fibroblasts (NRCFs) were inhibited by Ala treatment in vitro. The biomarkers of apoptosis were elevated in NRCFs induced by OGD, which were attenuated after treating with Ala in vitro. The enhancement of oxidative stress in the heart of MI mice or in the NRCFs treated with OGD was suppressed by treating with Ala in vivo and in vitro. These effects of Ala were reversed by tBHP, an exogenous inducer of oxidative stress in vitro. These results demonstrated that Ala could alleviate cardiac dysfunction and attenuate cardiac fibrosis via inhibition of oxidative stress.

**Supplementary Information:**

The online version contains supplementary material available at 10.1186/s13062-022-00338-6.

## Introduction

In adult mammals, left ventricular (LV) remodeling resulted from myocardial infarction (MI)-induced loss of cardiomyocytes and subsequent collagen deposition may serve as a structural and functional basis for the occurrence and progression of heart failure (HF) [1, 2]. Despite medical breakthroughs in interventional and pharmacological treatments to restore blood flow and improve myocardial cell energy metabolism, HF caused by MI still remains one of the progressive and fatal clinical diseases with the highest mortality worldwide, which ranks it among the major public health problems [3, 4]. Thus, more therapeutic agents required to be designed to exert a cardioprotective effects against MI-induced acute and chronic cardiac damage based on a more thorough understanding of the mechanisms underlying HF.

There is growing recognition and experimental evidence that the circulating renin–angiotensin–aldosterone system (RAAS), as a coordinated hormonal cascade, is critically important in regulating cardiovascular system under different physiological and pathological conditions [5]. As the most potent bioactive molecule involved in the classic angiotensin-converting enzyme-Angiotensin II-Angiotensin II type 1 receptor (ACE-AngII-AT1R) axis, AngII ensures the development of pathological cardiac remodeling upon myocardial infarction (MI) [6]. Ang-(1-7) is demonstrated to oppose the harmful effects of AngII/AT1R axis via binding to the Mas receptor [7]. Briefly, the introduction of Ang-(1-7) has been reported to improve myocardial tissue damages by inhibiting oxidative stress or alleviating inflammatory response and apoptosis under different pathological conditions, including hypoxia or ischemia/reperfusion (I/R) stimulation [8–10], which may partially explain the mechanisms underlying the therapeutic effects of ACE inhibitors (ACEIs) and AT1-receptor blockers (ARBs) [11].

The heptapeptide Ala-Arg-Val-Tyr-Ile-His-Pro (named as alamandine), a novel effector molecule of the RAAS protective arm, has the similar chemical structure of Ang-(1-7) with only one amino acid residue difference [12]. Alamandine (Ala) can be generated from the direct decarboxylation of Ang-(1-7) or from ACE2-mediated hydrolysis of angiotensin A [13]. Interestingly, the previous data on animals showed that Ala exerts similar antihypertrophic and hypotensive actions to Ang-(1-7) [14, 15]. Besides, Ala was reported to counterregulate pressure overload or AngII-induced cardiac remodeling via interacting with Mas-related G protein-coupled receptor member D (MrgD) [16, 17]. Also, Ala was shown to protect hearts from I/R injury [18].

The occurrence of oxidative stress which defined as excess peroxides and antioxidant deficit is consistent with the appearance of HF subsequent to MI [19]. Thus, the balance of ROS production and removal is essential for the redox state and homeostasis in the heart [20]. The maladaptive myocardial remodeling occurred in HF may be resulted from myocyte damage-induced enhanced production of ROS [21]. Also, the increased levels of ROS detected in heart tissue and plasma were even reported to be closely related to the severity of cardiac dysfunction in patients with HF [22]. Moreover, MI-induced accumulation of mitochondrial ROS was associated with cardiac fibroblasts activation [23], suggesting that cardiac fibroblasts are potential cellular targets for antioxidant therapies in HF. Notably, the present studies reported that Ala could substantially protect against organ fibrosis via inhibiting the production of ROS in pressure overload-induced cardiomyocytes, AngII-induced hepatocytes, and I/R-induced NRK52E cells [17, 24, 25]. However, no prior studies have examined the therapeutic effects of Ala on MI-induced HF and the subsequent cardiac fibrosis.

Thus, we addressed this in our present study via investigating the effects of Ala on HF and the related cardiac fibrosis, and further to probe the underlying molecular mechanisms.

## Materials and methods

### Ethics approval and animal care

Eight-week-old male Sprague–Dawley (SD) rats (Vital River Biological Co., Ltd, Beijing, China) were employed. All procedures of animal experiments were conducted in accordance with the Guide for the Care and Use of Laboratory Animals (NIH publication No. 85-23, revised 1996), and were approved by the Experimental Animal Care and Use Committee of Nanjing Medical University.

### Rat model of myocardial infarction

Male Sprague–Dawley rats (6–8 weeks old, 220–250 g) were purchased from Charles River Laboratories. Animals were anesthetized by intraperitoneal injection of sodium pentobarbital, then intubated and ventilated by a small animal ventilator (Model 680; Harvard Apparatus). Determine the tidal volume (1.5–2.5 mL) based on the respiratory rate and body weight of each animal. Body temperature is maintained by a heating pad. After opening the thoracic cavity and pericardium, the left anterior descending coronary artery (LAD) was non-invasively sutured by passing a 7-0 silk suture beneath the vessels and surrounding myocardium. The thoracotomy is then closed. Sham group was generated by the same process without LAD ligation. The thoracic cavity is closed layer by layer and regularly disinfected to prevent infection.

### Mice model of myocardial ischemia/reperfusion (I/R)

The mice in the I/R group and I/R + Ala group were subjected to LAD coronary artery ischemia for 30 min as we described above. Then, the ligature was released to induce reperfusion for 2 h [26, 27]. Meanwhile, the mice in the I/R + Ala group received tail intravenous injection of Ala (1 μM/kg) as we previously report [28]. Mice in the sham group only underwent a left thoracotomy. After that, mice were euthanized and harvested.

### Infarct size measurement

The mice hearts collected from different groups were frozen at − 20 °C for 30 min. Then, they were sectioned horizontally at 2–3 mm thickness. Following that, the hearts sections were incubated in 2% (w/v) 2,3,5-triphenyltetrazolium chloride (TTC) solution at 37 °C electric-heated water bath for 20 min. After formalin fixation, the sections were imaged. The infarct size identified by the white color area was determined by Image J software (MD, USA).

### Cell extraction and culture

Hearts were surgically excised from neonatal rats within 3 days of age, and myocardial tissue was cut into small pieces and incubated with 1.2 mg/mL pancreatin and 0.14 mg/mL collagenase (Gibco, Shanghai, China) at 37 °C. A series of digestions were performed in D-Hanks solution. After centrifugation, cells were suspended in Dulbecco's modified Eagle's medium (DMEM; Gibco, Shanghai, China) containing 20% fetal bovine serum, 100 U/mL penicillin, and 100 μg/mL streptomycin. The dissociated cells were pre-plated at 37 °C for 1 h, and cardiomyocytes were isolated by rapid adhesion of cardiac fibroblasts. Subsequently, cardiomyocytes were collected, and plated onto gelatin-coated dishes. The cardiac fibroblasts were cultured in the DMEM complete medium. Place the cells in a 37 °C 5% CO_2_ incubator for subsequent experiments.

The culture medium was replaced with serum-free and glucose-free DMEM in the presence or absence of Ala (10^−6^ mol/L) when the cardiac fibroblasts in the oxygen–glucose deprivation (OGD) or OGD + Ala group reached 80% confluence. Then, the dishes containing cells were transferred to a hypoxic chamber containing 5% CO_2_, 94% N_2_, and 1% O_2_ as previously report [29]. After stimulation for 8 h at 37 °C as previously report [30], the cells were then harvested and analyzed. In some experiments, cells were preliminary incubated with 5 mmol/l N-acetylcysteine (S1623, Selleck Co., China), and 200umol/l tert-Butyl hydroperoxide (tBHP, B802372, Medchemexpress Co., China) for 2–3 h prior to OGD stimulation.

### Western blot analysis

Rat hearts were removed, immediately frozen in liquid nitrogen, and stored at − 80 ℃ until further use. The cardiac tissues or cultured cells were sonicated in Radio Immunoprecipitation Assay [31] lysis buffer and homogenized. The debris was removed, and the supernatant was collected after centrifugation at 12,000×*g* for 10 min at 4 °C. The protein concentration was then determined at 562 nm using the Pierce™ BCA Protein Quantitation Kit (Invitrogen, Shanghai, China) in a microplate reader. About 30–50 μg of protein were separated by electrophoresis, and according to the molecular weight of the target protein, the labeled bands in the desired molecular weight range were separated. A polyvinylidene fluoride membrane (PVDF) (Millipore) was cut to the size of a separation gel, immersed in anhydrous methanol for 5 min, covered with the gel, and transferred at a constant current of 300 mA for 120 min. and blocking Tris-buffered saline in bovine serum albumin (BSA). Membranes were mixed with collagen I, (1:1000; No.14695-1-AP; Proteintech Co., Wuhan, China), α-SMA (1:1000; No.14395-1-AP; Proteintech), TGF-β (1:1000; No.21898-1-AP; Proteintech), CC3 (1:1000; #9664; Cell Signaling Technology), C3 (1:1000; #9662; Cell Signaling Technology), Bcl2 (1:1000; #3498; Cell Signaling Technology), Bax (1:1000; #5023; Cell Signaling Technology), and GAPDH (1:1000; AF0006; Beyotime Biotechnology Co., Shanghai, China) overnight at 4 °C with gentle shaking. Goat anti-rabbit IgG-HRP (1:2000, ab6721) was added as secondary antibody and incubated for 2 h at 37 °C with gentle shaking. The PVDF membrane was then rinsed three times (5 min each) with TBST at room temperature. Next, chemiluminescent reagent (ECL) (Pierce) was evenly spread on the PVDF membrane. Images were analyzed using the Image-Pro Plus software.

### Reverse transcription-quantitative polymerase chain reaction (RT‐qPCR)

Total RNA was extracted from myocardial tissue using Trizol (Invitrogen, Shanghai, China). Total RNA was synthesized into cDNA by reverse transcription in 10 μL reactions according to the instructions of PrimeScript™ RT Master Mix Kit (TaKaRa Biomedical Technology, Beijing, China). Primers for genes were designed and synthesized by Genscript (Table 1). Subsequently, mRNA was assessed by SYBR Green I fluorescence. All samples were amplified in triplicate for 40 cycles in 384-well plates. The 2-ΔΔCt method was used to determine the ratio of target gene expression between the experimental group and the control group.

**Table 1:** List of utilized primers for qRT-PCR

Gene	Species	Forward primer	Reverse primer
α-SMA	Rat	GCATCCACGAAACCACCTA	CACGAGTAACAAATCAAAGC
TGF-β	Rat	TCTGCATTGCACTTATGCTGA	AAAGGGCGATCTAGTGATGGA
Collagen I	Rat	GCTCCTCTTAGGGGCCACT	CCACGTCTCACCATTGGGG
GAPDH	Rat	GGCACAGTCAAGGCTGAGAATG	ATGGTGGTGAAGACGCCAGTA

*α-SMA* α-smooth muscle actin, *TGF-β* transforming growth factor-β, *GAPDH* glyceraldehyde-3-phosphate dehydrogenase

### TUNEL staining assay

The TUNEL staining assay was performed according to the One Step TUNEL Apoptosis Assay Kit (Beyotime, Shanghai, China). First, the cells were fixed with 4% paraformaldehyde for 15 min at room temperature, and permeabilized with 0.5% Triton X-100 for 5 min to rupture the membrane. Then, the cells were incubated with the TUNEL reaction mixture provided with the staining kit for 60 min at 37 °C in the dark before staining with DAPI. Finally, the stained cells were scanned and imaged under a fluorescence microscope.

### Measurement of intracellular reactive oxygen species (ROS) levels

Intracellular ROS levels were measured using the assay kit (Beyotime Biotechnology, Shanghai, China); 2′,7′-dichlorofluorescein diacetate (DCFH-DA) is easily oxidized by intracellular ROS to fluorescent dichloride Fluorescein (DCF) is the main component of this kit. Briefly, NRCF cells were seeded in 96-well plates as described above and divided into different groups required for the experiment. Then, cells were incubated with DCFH-DA in the kit for 20 min at 37 °C and co-incubated with DAPI for 5 min. The images were finally captured under a fluorescence microscope.

### Statistical analyses

GraphPad Prism 7.0 (GraphPad Software Inc., San Diego, CA, USA) was applied to present all the data as mean ± standard error of the mean (SEM). All the data was analyzed for normality using the D'Agostino & Pearson's test. The statistical significance for multigroup comparisons was evaluated by one-way analysis of variance (ANOVA), followed by Bonferroni’s post-hoc tests.

## Results

### Ala alleviated MI-induced cardiac dysfunction

**Fig. 1:**
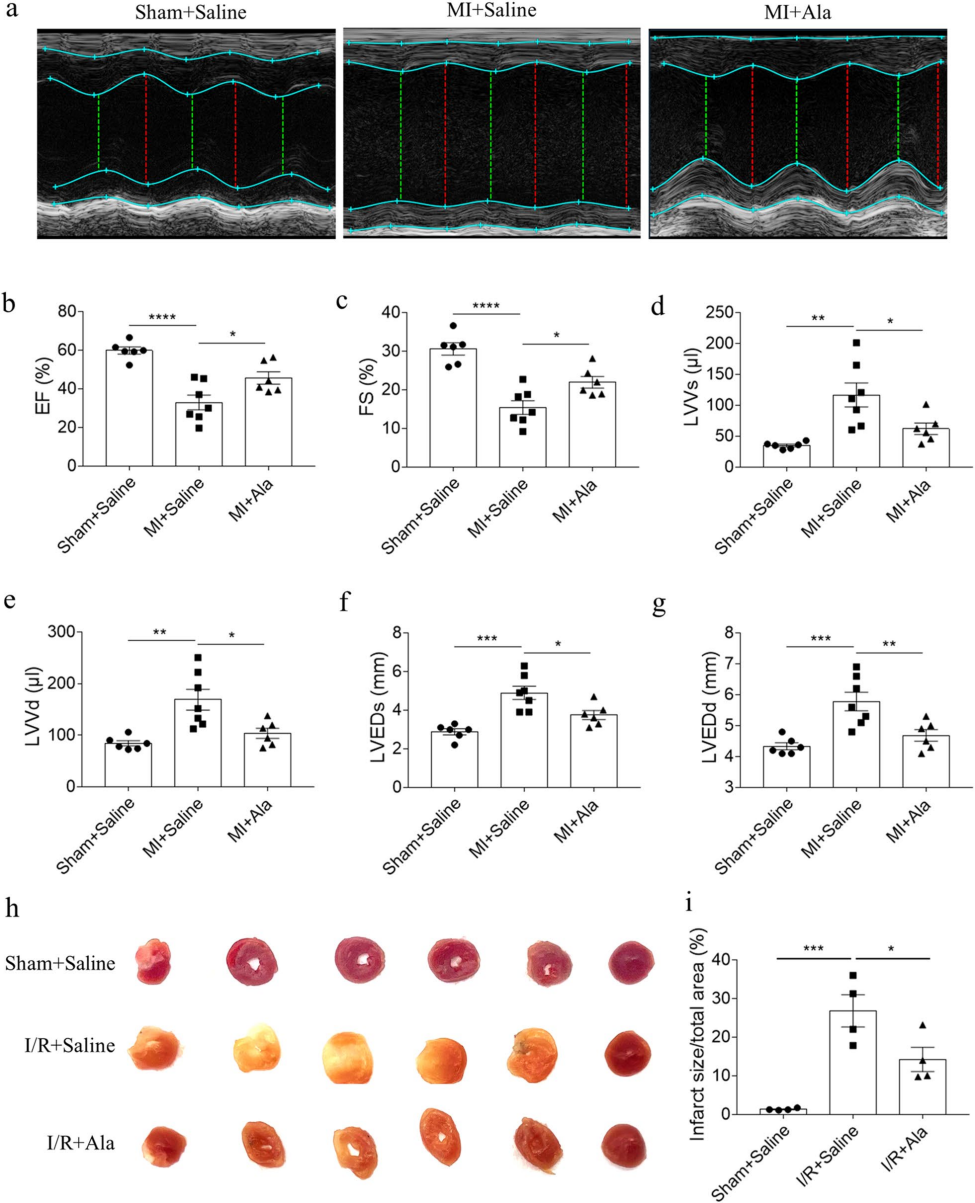
Ala alleviated MI-induced cardiac dysfunctionn. **a**–**g** Ala administration reversed the decreases of EF and FS, and the increases of LVVs, LVVd, LVEDs and LVEDd in MI-induced HF mice. N = 6 in Sham + Saline and MI + Ala groups, and N = 7 in MI + Saline group. **h**–**i** Ala administration alleviated the cardiac damage of I/R mice. N = 4 for each group. The results are expressed as mean ± SEM

Heart failure was induced by MI in mice, and Ala was injected two week after MI lasting two weeks. Representative images of echocardiography in the three groups were shown (Fig. 1a). The EF and FS of LV were reduced in MI mice, which were reversed after injection of Ala (Fig. 1b, c). The LVVs, LVVd, LVEDs and LVEDd of LV were elevated in MI mice. These increases were attenuated after treating with Ala in vivo (Fig. 1d–g). Ala administration significantly alleviated cardiac damage of I/R mice (Fig. 1h, i).

### Ala alleviated MI-induced cardiac fibrosis

**Fig. 2:**
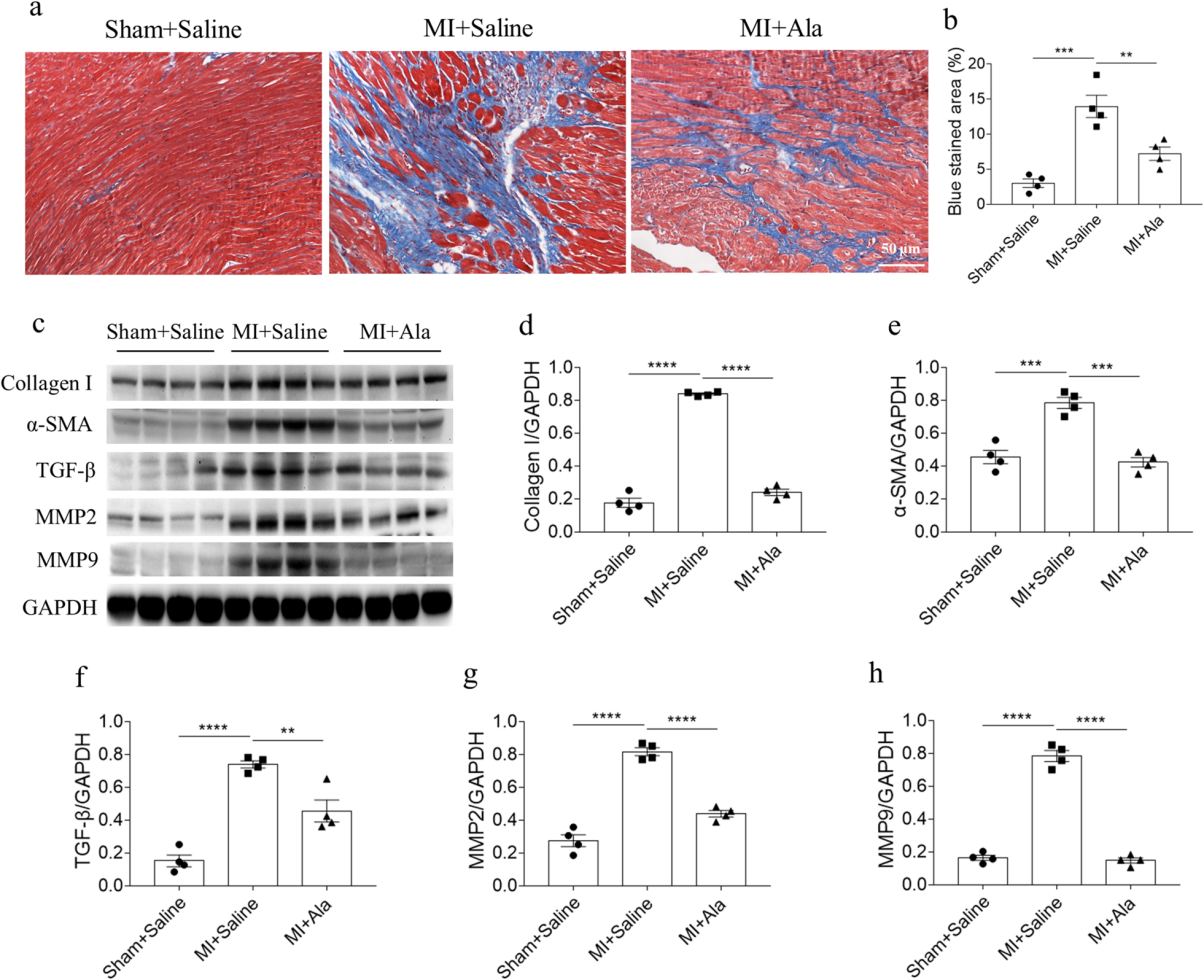
Ala alleviated MI-induced cardiac fibrosis. **a**, **b** The fibrosis of heart was alleviated after administration of Ala in MI-induced HF mice. **c–h** The increases of collagen I, α-SMA, TGF-β, MMP2 and MMP9 in the heart of MI-induced HF mice were suppressed after administration of Ala. N = 6 in each group. The results are expressed as mean ± SEM

MI-induced HF produced cardiac fibrosis. The fibrosis of heart was enhanced in MI-induced HF mice, and this enhancement was significantly attenuated by Ala treatment via detecting with masson staining (Fig. 2a). The biomarkers of cardiac fibrosis were detected in the next research. The protein levels of collagen I, α-SMA, TGF-β, MMP2 and MMP9 were increased in the heart of MI mice, which were suppressed via treating with Ala in vivo (Fig. 2c–h).

### Ala alleviated OGD-induced fibrosis and apoptosis of NRCFs

**Fig. 3:**
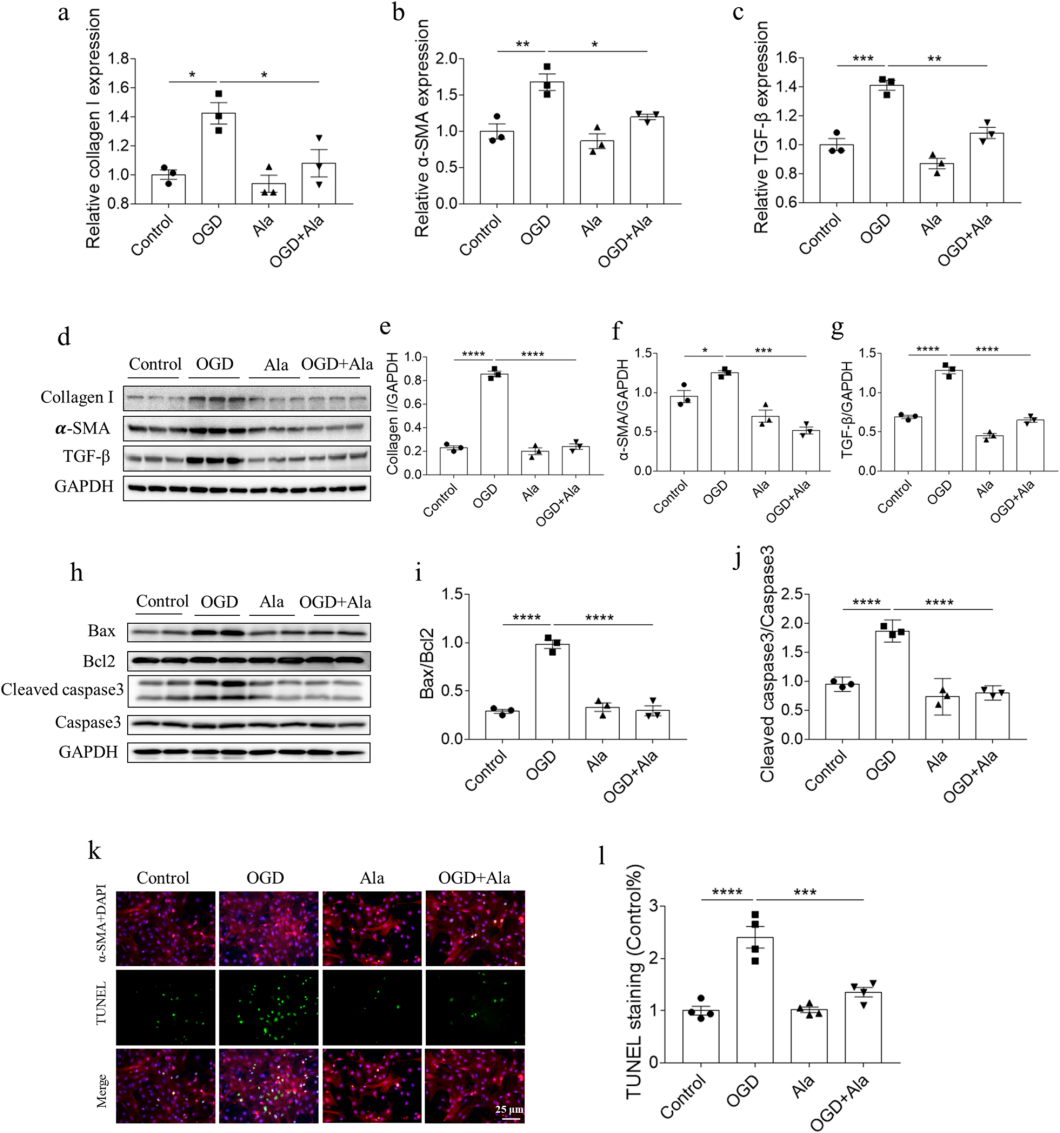
Ala alleviated OGD-induced fibrosis and apoptosis of NRCFs. **a**–**c** Ala inhibited the increases of collagen I, α-SMA and TGF-β mRNA induced by OGD in NRCFs. **d**–**g** Ala inhibited the increases of collagen I, α-SMA and TGF-β proteins induced by OGD in NRCFs. **h**–**j** Ala inhibited the increases of Bax/Bcl2 and cleaved caspase3/caspase3 induced by OGD in NRCFs. **k**, **l** Ala inhibited the increase of TUNEL positive cells induced by OGD in NRCFs. N = 3 in each group (**a**–**j**), and N = 4 in each group (**k**, **l**). The results are expressed as mean ± SEM

The mRNA levels of collagen I, α-SMA and TGF-β were increased in OGD-treated NRCFs, and these increases were inhibited after Ala treatment in vivo (Fig. 3a–c). Ala also suppressed the increases of collagen I, α-SMA and TGF-β proteins induced by OGD in NRCFs (Fig. 3d–g). The biomarkers of apoptosis were detected to determine the effect of Ala on OGD-induced apoptosis of NRCFs. The results showed that the increases of Bax/Bcl2 and cleaved caspase3/caspase induced by OGD in NRCFs were suppressed by Ala treatment (Fig. 3h–j). In addition, the TUNEL staining was performed to further evaluate the role of Ala in OGD-induced NRCFs apoptosis. We found that Ala treatment significantly attenuated the increase of TUNEL positive cells induced by OGD in vitro (Fig. 3k, l).

### Ala alleviated OGD-induced oxidative stress in NRCFs

**Fig. 4:**
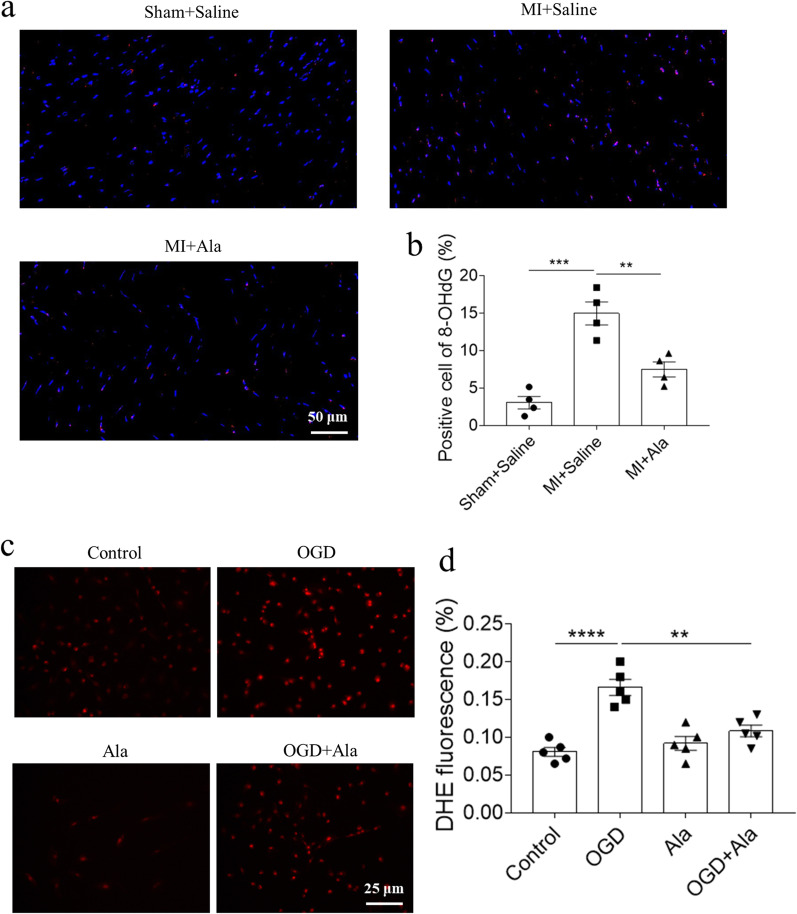
Ala alleviated OGD-induced oxidative stress in NRCFs. **a**, **b** Ala inhibited the increase of 8-OHdG positive cells in the heart of MI-induced HF mice. **c**, **d** Ala inhibited the increase of ROS level induced by OGD in NRCFs. N = 4 in each group (**a**, **b**), and N = 5 in each group (**c**, **d**). The results are expressed as mean ± SEM

MI-induced oxidative stress may deteriorate cardiac repair, resulting in the pathological cardiac remodeling [32]. Thus, we further investigated the role of oxidative stress in the protective effects of Ala on OGD-induced NRCFs. 8-OHdG level and DHE staining were detected to evaluate the oxidative stress. We found that the number of 8-OHdG positive cells was increased in the heart of MI mice, which was suppressed after Ala administration (Fig. 4a, b). DHE staining showed that ROS level was elevated in OGD-treated NRCFs, and this increase was attenuated after administration of Ala in vitro (Fig. 4c, d).

### Oxidative stress enhancement reversed the effects of Ala in NRCFs

**Fig. 5:**
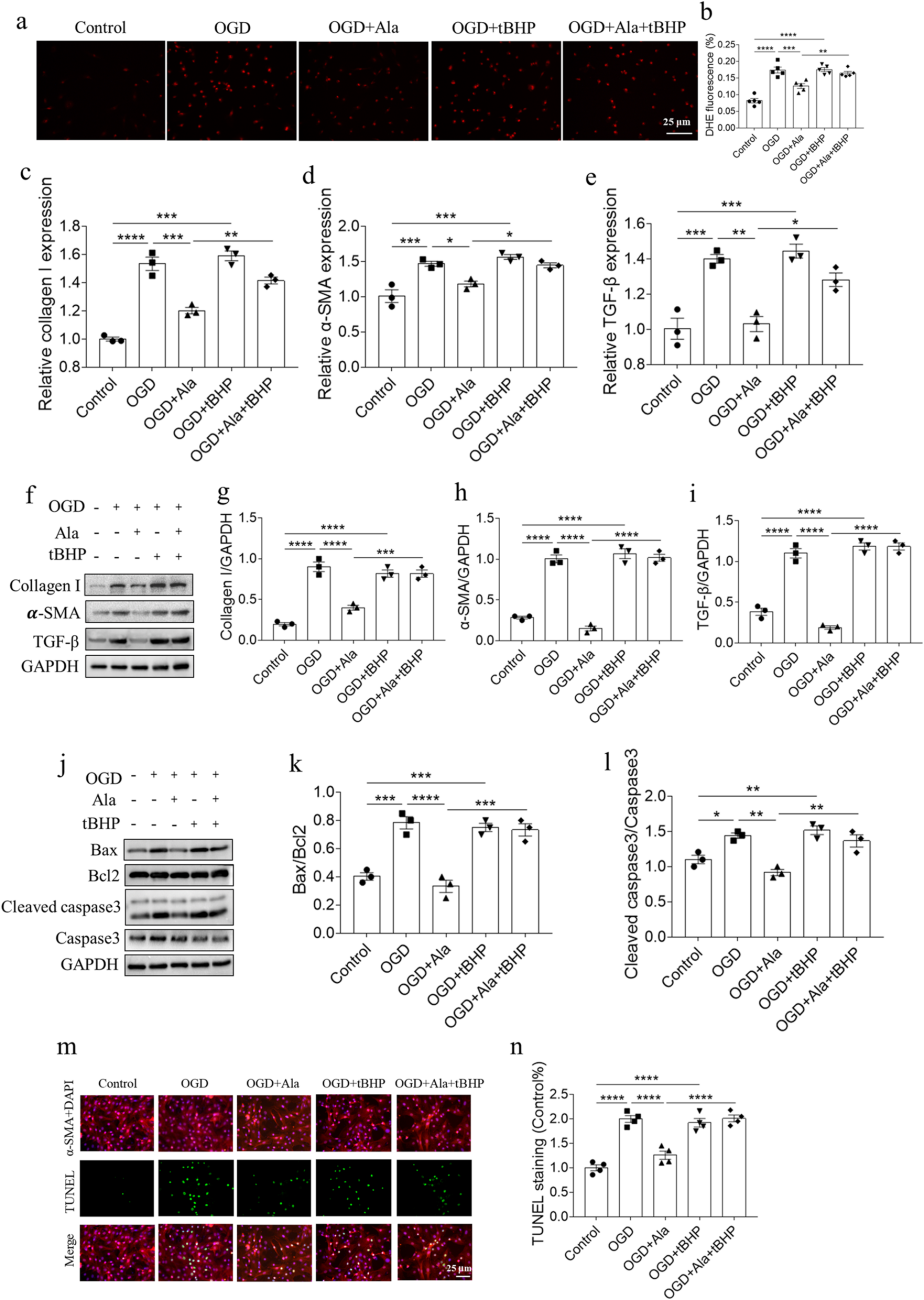
Oxidative stress enhancement reversed the effects of Ala in NRCFs. **a**, **b** tBHP reversed the inhibiting effect of Ala on OGD-induced ROS increase in NRCFs. **c**–**e** tBHP reversed the inhibiting effect of Ala on OGD-induced the increases of collagen I, α-SMA and TGF-β mRNA in NRCFs. **f**–**i** tBHP reversed the inhibiting effect of Ala on OGD-induced the increases of collagen I, α-SMA and TGF-β proteins in NRCFs. **j**–**l** tBHP reversed the inhibiting effect of Ala on OGD-induced the increases of Bax/Bcl2 and cleaved caspase3/caspase3 in NRCFs. **m**, **n** tBHP reversed the inhibiting effect of Ala on OGD-induced the increase of TUNEL positive cells in NRCFs. N = 5 in each group (**a**, **b**), N = 3 in each group (**c**, **l**), and N = 4 in each group (**m**, **n**). The results are expressed as mean ± SEM

tBHP is an exogenous inducer of oxidative stress. DHE staining showed that the inhibiting effect of Ala on OGD-induced ROS increase was reversed by tBHP in NRCFs (Fig. 5a). The attenuating effects of Ala on the increases of collagen I, α-SMA and TGF-β mRNA in NRCFs induced by OGD were recovered after treatment with tBHP (Fig. 5c–e). These effects of Ala were further proved by detecting the protein levels of collagen I, α-SMA and TGF-β (Fig. 5f–i). Similarly, the inhibiting effects of Ala on the increases of Bax/Bcl2 and cleaved caspase3/caspase3 induced by OGD in NRCFs were reversed by tBHP treatment (Fig. 5j–l). In addition, the attenuating role of Ala in the increase of TUNEL positive cell induced by OGD in NRCFs was reversed by administration of tBHP in vitro (Fig. 5m, n).

NAC is a well-known antioxidant. DHE staining showed that NAC suppressed the increase of ROS level in NRCFs induced by OGD (Additional file 1: Figure S1a, b). The increases of collagen I, α-SMA and TGF-β mRNA in OGD-treated NRCFs were attenuated via NAC treatment (Additional file 1: Figure S1c–e). The increases of collagen I, α-SMA and TGF-β protein in NRCFs induced by OGD were inhibited by treating with NAC (Additional file 1: Figure S1f–i). The increases of Bax/Bcl2 and cleaved caspase3/caspase induced by OGD in NRCFs were inhibited by NAC (Additional file 1: Figure S1j–l). NAC administration markedly suppressed the increase of TUNEL positive NRCFs induced by OGD in vitro (Additional file 1: Figure S1m, n).

## Discussion

As the main cellular constituent in the myocardium, cardiac fibroblasts can differentiate into synthetic myofibroblasts with high proliferative capacity [33]. The persistent myofibroblast activation may aid with cardiac restoration and healing manifested as activated matrix-preserving program and cardiac fibrosis [34]. Several commonly therapeutic approaches for CVDs exert pleiotropic effects on cardiac fibroblasts [35], suggesting that cardiac fibroblasts could become a promising target for HF subsequent to MI.

OGD was found to induce ischemic heart injury by triggering a complex pathological process that included excessive ROS production and extrinsic and intrinsic apoptosis in cardiac cells [36, 37]. Thus, here in our study, OGD was applied to the cardiac fibroblasts to construct a cellular model of ischemic heart injury in vitro. Indeed, our data showed that OGD induced fibrosis in cardiac fibroblasts in vitro.

Till now, most agents designed targeting different pathogenesis of HF have been introduced to play preventive protective role in MI-induced cardiac remodeling via pre-administration before or at the onset of the injure, while their efficacy gradually diminishes as the course of the disease progresses [38, 39]. The sudden onset of MI and its extreme narrow therapeutic window enable physicians rarely to accurately predict and timely administer drugs, making it considered challenged to translate these therapeutic strategies which need pre-injury administration into the clinical realm [40]. Thus, it is of great interest to develop novel strategy that is time-insensitive to the ischemic insult [41].

Not only the circulating AngII and aldosterone, but also local AngII produced by MI stimulation accentuates overactive cardiac fibrosis by promoting myofibroblast differentiation and matrix synthesis in the infarcted myocardium [42, 43]. The cardiac RAAS was well-described to be a complex endocrine system that carries out regulatory actions in the development and pathogenesis of muscular (vascular and cardiac) hypertrophy and fibrosis in various pathological states, such as MI [42, 44]. The fact that MI-induced imbalance of RAAS could lead to the resultant cardiac remodeling makes blocking RAAS a promising treatment for an overwhelming number of cardiovascular and renal complications following MI [45]. In this context, extensive experimental evidence and clinical trials have suggested the clinical practice of ACEI or ARB in the treatment of chronic HF [46, 47].

Relatively, there is still a lack of understanding towards the contribution of AT2 receptors in cardiac remodeling. Despite the barely detection of AT2 receptors in the infarcted rat myocardium [48], AT2 receptor was still reported to favorably influence post-MI cardiac wound healing and repair [49]. Activation of AT2R, which has the opposing effects against AT1R-mediated cardiac hypertrophy, has been revealed to reduce the early mortality and attenuate myocardial remodeling following MI [50–52].

Recently, bone marrow mesenchymal stem cell-derived exosomes (BMSC-EX) was found to form sustained myocardial protection against acute MI partly through accelerating the conversion of AngII to Ang-(1-7) [53]. Olmesartan [30] has also been reported to attenuate myocardial remodeling via activating ACE2-Ang-(1-7)-MasR axis [54], suggesting new strategy utilizing ACE2-Ang-(1-7)-MasR axis as a promising pharmacological target in MI.

The introduction of Ang-(1-7) appears to afford improved effects on oxygenation while attenuating inflammation and fibrosis [55]. Studies on animals indicated that Ang-(1-7) alone could protect significantly against High glucose (HG) or I/R-stimulated cardiac injure as an inhibitor of oxidative stress [56, 57]. Also, Ang-(1-7) may potentiate the cardioprotective effects of ischemic preconditioning (IPC) or other conventional approaches on combating mitochondrial dysfunction, reactive oxygen species (ROS) production and apoptosis in CVDs [55, 56]. The significant anti-apoptotic and anti-oxidative actions of Ang-(1-7) during cardiac dysfunction provide a new direction for the clinical treatment of MI-induced heart failure [10, 58, 59].

Notably, previous study showed increased Ang-(1-7) expression in the myocytes of rats with HF subsequent to MI [60]. Another study indicated circulating rather than cardiac Ang-(1-7) to be beneficial after MI [61]. Moreover, Ang-(1-7) or Ang-(1-7) receptor Mas agonist AVE-0991 could present a cardioprotective role in the development of HF and cardiac remodeling post-MI [62, 63]. Also, Ang-(1-7) oral treatment after MI may improve cardiac remodeling [64]. Above studies characterized Ang-(1-7) as a potential diagnostic and therapeutic tool in MI. The apparent similarity of biological effects between Ala and Ang-(1-7) aroused interest in developing Ala as a promising therapeutic target for HF [13–15]. Also, Ala was shown to protect hearts from I/R injury [18]. Cardiac myofibroblasts were reported as not only the major non-myocyte cells participated in the post-MI infarct healing and the subsequent cardiac remodeling, but also the major cells expressing AT1R upon MI [35, 65]. It is noteworthy that the activated infarct myofibroblasts that survive the ischemic injure may generate AngII in the infarcted myocardium which facilitates heart failure progression [65], prompting whether Ala, a member in the RAAS protective arm could alleviate HF. Consistent with our supposition, our present data proved that the introduction of Ala improved HF subsequent to MI in mice. To our knowledge, we demonstrated for the first time that Ala attenuated MI-induced cardiac dysfunction and OGD-induced cardiac fibrosis and apoptosis via inhibiting oxidative stress in vivo and in vitro, respectively.

In addition to MI, myocardial I/R, which is more closely paralleled the clinical scenario, may also do harm to the heart tissues [66]. In order to provide a more accurate model of I/R injury than in vitro OGD, although previous studies have reported the cardioprotective effects of Ala against acute and chronic myocardial I/R injury, respectively [18, 67], we still investigated the role of Ala in I/R-induced myocardial ischemic injury. We also established the I/R mice model with 30 min of ischemia followed by 2 h of reperfusion as previously report [26, 27]. Indeed, we found that Ala diminished I/R-induced infarct size of hearts, which may further verify the beneficial role of Ala in hypoxic-induced cardiac damage.

Myocyte apoptosis in response to ischemic injure was considered as one of the mechanisms by which HF progressed [68, 69]. Initially, the collagen-secreting cardiac fibroblasts would undergo apoptosis upon MI-induced acute myocardial injury [70]. Then, the remained activated fibroblasts within cardiac scars may potentiate and perpetuate the pathophysiologic processes of cardiac replacement fibrosis and hypertrophy [71, 72]. The exosomes derived from hypoxia-induced cardiomyocytes was found to promote cardiac fibroblasts apoptosis [73]. Thus, we further investigated the capability of Ala in suppressing cell apoptosis upon OGD stimulation. Our data showed that Ala reduced OGD-induced apoptosis in cardiac fibroblasts and MI-induced apoptosis in heart tissues in vivo and in vitro. Notably, the previous study reported that OGD was able to increase apoptosis in cardiac fibroblasts, thereby inducing cardiac injury [74], which was in accordance with our observations. Interestingly, another study, which may contradict the recognized viewpoint that anoxia could stimulate cardiac fibroblast proliferation, has observed that I/R or ischemia stimulation could induce significant apoptosis in cardiac fibroblasts, which could be explained by the differences in methodology (anoxia vs hypoxia) of in vitro experiments in these two studies [30, 75]. Indeed, our model of OGD that included hypoxic stimulation, nutrient deprivation and glycolysis inhibition was more deleterious than anoxia itself.

Pathological stimulation-evoked oxidative stress could active various signaling pathways involved in the cardiomyocyte apoptosis, including caspase-3 [12, 31]. Also, oxidative stress was proposed to trigger myocytes apoptosis in ischemic heart diseases, which is mainly manifested by the upregulation of proapoptotic proteins, including Bax and caspases [76, 77].

The substantial evidence has shown that MI-induced oxidative disruptions to the myocardium could deteriorate cardiac repair phase and further contribute to adverse cardiac outcomes, including cardiac fibrosis and malfunction [32]. The imbalanced redox state induced by ischemia or hypoxia-stimulated overproduction of ROS may be manifested as increased production of lipid peroxides, such as MDA, a significant hallmark of oxidative stress [78]. The ROS implicated in CVDs generated mainly from mitochondria [79]. Mitochondrial damage was reported to be related to the increased generation of ROS when oxygen availability decreases [80]. Besides, the deranged mitochondria with relatively excess reactive oxygen species (ROS) production were found in the peripheral blood mononuclear cells (PBMCs) isolated from patients with chronic HF, suggesting that oxidative stress may be one of the most important mechanisms responsible for the occurrence and progression of cardiac remodeling in HF [81, 82]. Accordingly, it was found that the anti-oxidant, vitamin E or probucol, could markedly prevent post-MI cardiac remodeling and hemodynamics, while scavenging oxidative stress [72, 83]. Also, Ala co-therapy could improve LPS or Doxorubicin (DOX)-induced cardiac dysfunction by its antioxidant and anti-apoptotic activities [28, 84]. The inhibitory effects of non-secretory renin in ODG-induced oxidative stress and cardiomyoblasts apoptosis provides us further incentives to explore the role of oxidative stress in the anti-apoptotic and anti-fibrotic effects of Ala in OGD-induced cardiac fibroblast [85]. In our study, Ala suppressed OGD-induced ROS production and MDA levels and apoptosis in cardiac fibroblasts. Then, the anti-fibrotic effects of Ala were enhanced by pre-treatment of ROS scavenger N-acetylcysteine in vitro, while that was reversed by ROS producing agent tBHP in vitro, suggesting that the effects of Ala functionally dependent on ROS level.

The understanding of the balance between the two branches of RAS under different physiopathological conditions furnish novel therapeutic targets that may mitigate the development of CVDs [86]. Previous studies have confirmed the cardioprotective effects of ACE2 activators and ACE inhibitors (ACEIs) against MI-induced cardiac injure [87–90]. Notably, ACEIs were found to increase circulating Ang-(1-7) levels [91]. Ang-(1-7) was confirmed to reverse AngII-induced cardiac injure through the Mas receptor [92]. Recently, increasing Ang-(1-7) have been considered to be a promising alternative mean to ACEIs or ACE2 activators that could improve cardiac function [93, 94]. Likewise, each of the components in the non-canonical branch of RAS, including Ang-(1-7), Ang-(1-9), ACE2, AT2R, and Ala, has also been shown to counteract the effects of AngII/AT1R axis [95]. Among them, Ala possessed its biological actions by binding to its endogenous receptor Mas-related G protein-coupled receptor member D (MrgD), which was blocked by the AT2R antagonist PD123319 or Mas/MrgD antagonist D-Pro(7)-angiotensin-(1-7) [12, 96]. To our minds, since Ala was formed from the hydrolysis of Ang A via ACE2 [97], it could be a viable alternative to well-established ACE2 activators in order to achieve higher Ala levels. Also, Ala may be a potential add-on therapy of patients with HF with reduced ejection fraction (HFrEF), which is yet to be supported by the results of more rigorous clinical randomized controlled trials. Especially, the difference of Ala concentration in peripheral blood or in situ heart tissues between patients with myocardial infarction and normal healthy subjects detected by liquid chromatography-mass spectrometry could provide more valuable basis for the clinical introduction of Ala for the treatment of HF post-MI.

## Conclusions

Overall, our current data sheds light on the cardioprotective effects of Ala in MI rats. Mechanistically, Ala could attenuate OGD-induced cardiac fibrosis and apoptosis via suppressing oxidative stress in vitro, which paving the way for developing it as a promising clinical treatment.

## Supplementary Information

**Additional file 1: Figure S1**. Antioxidant alleviated OGD-induced fibrosis and apoptosis of NRCFs. **a**, **b** NAC attenuated the increase of ROS production in NRCFs induced by OGD. **c**–**e** NAC inhibited the increases of collagen I, α-SMA and TGF-β mRNA induced by OGD in NRCFs. **f**–**i** NAC inhibited the increases of collagen I, α-SMA and TGF-β proteins induced by OGD in NRCFs. **j**–**l** NAC inhibited the increases of Bax/Bcl2 and cleaved caspase3 induced by OGD in NRCFs. **m**, **n** NAC inhibited the increase of TUNEL positive cells induced by OGD in NRCFs. N = 5 in each group (**a**, **b**), N=3 in each group (**c**–**l**), and N = 4 in each group (**m**, **n**). The results are expressed as mean ± SEM.

## Data Availability

Available upon requests.
