# Supplementary figures and images for: Alamandine alleviated heart failure and fibrosis in myocardial infarction mice
Source: Biol Direct. 2022 Sep 27;17:25. doi: 10.1186/s13062-022-00338-6 (PMC9516792; doi:10.1186/s13062-022-00338-6)

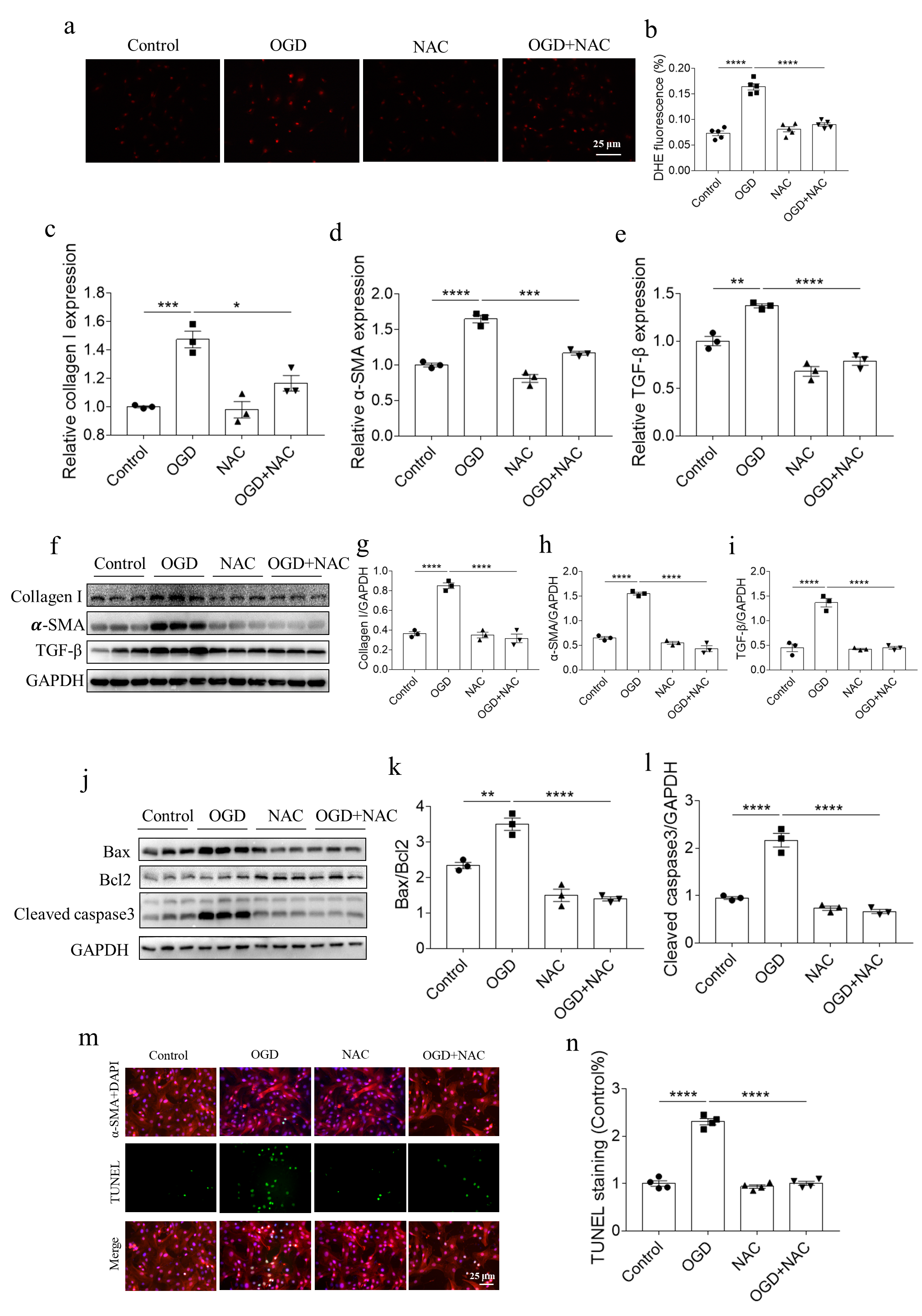

Supplement: Supplementary file 1 — Additional file 1: Figure S1. Antioxidant alleviated OGD-induced fibrosis and apoptosis of NRCFs. a, b NAC attenuated the increase of ROS production in NRCFs induced by OGD. c–e NAC inhibited the increases of collagen I, α-SMA and TGF-β mRNA induced by OGD in NRCFs. f–i NAC inhibited the increases of collagen I, α-SMA and TGF-β proteins induced by OGD in NRCFs. j–l NAC inhibited the increases of Bax/Bcl2 and cleaved caspase3 induced by OGD in NRCFs. m, n NAC inhibited the increase of TUNEL positive cells induced by OGD in NRCFs. N = 5 in each group (a, b), N=3 in each group (c–l), and N = 4 in each group (m, n). The results are expressed as mean ± SEM. [file 13062_2022_338_MOESM1_ESM.tif]
